# Nail Lacquer Containing *Origanum vulgare* and *Rosmarinus officinalis* Essential Oils and Biogenic Silver Nanoparticles for Onychomycosis: Development, Characterization, and Evaluation of Antifungal Efficacy

**DOI:** 10.3390/antibiotics13090892

**Published:** 2024-09-17

**Authors:** Sara Scandorieiro, Natalia Rodrigues de Oliveira, Monique de Souza, Lidiane Vizioli de Castro-Hoshino, Mauro Luciano Baesso, Gerson Nakazato, Renata Katsuko Takayama Kobayashi, Luciano Aparecido Panagio, Audrey Alesandra Stinghen Garcia Lonni

**Affiliations:** 1 Laboratory of Innovation and Cosmeceutical Technology, Department of Pharmaceutical Sciences, Center of Health Sciences, Hospital Universitário de Londrina, Robert Koch Avenue, 60, Londrina 86038-350, Brazil; nataliar805@gmail.com; 2 Department of Physics, Center of Exact Sciences, Universidade Estadual de Maringá, Colombo Avenue, 5790, Maringá 87020-900, Brazil; monikdsouza@hotmail.com (M.d.S.); lidiane.hoshino@gmail.com (L.V.d.C.-H.); mlbaesso@uem.br (M.L.B.); 3 Laboratory of Basic and Applied Bacteriology, Department of Microbiology, Center of Biological Sciences, Universidade Estadual de Londrina, Celso Garcia Cid Road, PR-445, Km 380, University Campus, Londrina 86057-970, Brazil; gnakazato@uel.br (G.N.); kobayashirkt@uel.br (R.K.T.K.); 4 Laboratory of Medical Mycology and Oral Microbiology, Department of Microbiology, Center of Biological Sciences, Universidade Estadual de Londrina, Celso Garcia Cid Road, PR-445, Km 380, University Campus, Londrina 86057-970, Brazil; lapanagio@uel.br

**Keywords:** nail mycosis, antifungal nail lacquer, topical treatment, green nanotechnology, essential oils, *Trichophyton* spp., *Microsporum* spp.

## Abstract

Onychomycosis is a common fungal nail infection for which new antifungals are needed to overcome antimicrobial resistance and the limitations of conventional treatments. This study reports the development of antifungal nail lacquers containing oregano essential oil (OEO), rosemary essential oil (REO), and biogenic silver nanoparticles (bioAgNPs). The formulations (F) were tested against dermatophytes using agar diffusion, ex vivo nail infection, and scanning electron microscopy techniques. They were evaluated for their pharmacotechnical characteristics and by FTIR-PAS to assess permeation across the nail. F-OEO and F-OEO/bioAgNPs were promising candidates for the final nail lacquer formulation, as they permeated through the nail and showed antifungal efficacy against dermatophytes-contaminated nails after 5 days of treatment. Treated nails exhibited decreased hyphae and spores compared to the untreated control; the hyphae were atypically flattened, indicating loss of cytoplasmic content due to damage to the cytoplasmic membrane. The formulations were stable after centrifugation and thermal stress, maintaining organoleptic and physicochemical characteristics. Both F-OEO and F-OEO/bioAgNPs had pH compatible with the nail and drying times (59–90 s) within the reference for nail lacquer. For the first time, OEO and bioAgNPs were incorporated into nail lacquer, resulting in a natural and nanotechnological product for onychomycosis that could combat microbial resistance.

## 1. Introduction

The nail plate is a rigid structure made of a hard substance called keratin that protects the ends of the fingers and toes [1]. Several nail disorders are seen in clinical practice; they can be associated with nutritional deficiencies, aging, trauma, medication use, and hematological or endocrinological causes, among others [1,2]. Infectious diseases such as onychomycosis, pulmonary tuberculosis, and syphilis can also cause damage to the nail plate. Onychomycosis is the most common nail disorder [2,3,4].

Onychomycosis is a fungal infection that primarily affects the toenails. It is caused by dermatophytes, yeasts, and saprophytic molds. The most common etiological agent is *Trichophyton rubrum*, a species of dermatophyte fungus. When dermatophytes cause this nail infection, this condition is called tinea unguium. The prevalence of onychomycosis ranges from 1 to 8%, and its incidence is increasing. The disease is associated with genetic predisposition [4].

Dermatophytes are free-living in the environment, but they are keratinophilic fungi that can infect keratinized tissue such as the nails. Onychomycosis is often preceded by an asymptomatic hyperkeratotic tinea pedis. The use of shoes creates an environment (warm, dark, and humid) that favors fungal growth; the traumatic pressure on the nail unit can disrupt the hyponychial seal and facilitate the access of dermatophytes to the nail bed. Dermatophytes produce keratinases that allow infection to spread through the tissue. The acute lesion involves spongiosis, acanthosis, papillomatosis with edema, and hyperkeratosis. Inflammation occurs at the site of infection, and the infection may progress to a chronic state characterized by extensive compact hyperkeratosis, hypergranulosis, acanthosis, and papillomatosis with infiltrate [4]. The infected nail begins to present a dystrophic appearance, showing color change, onycholysis, and hyperkeratosis, in addition to causing pain and discomfort to the individual [4,5].

In addition to the physical discomfort, it is important to emphasize the substantial psychological impact associated with onychomycosis. The appearance of diseased nails may affect the self-esteem of the patients, generating feelings of embarrassment and anguish and even leading to social isolation, thus profoundly damaging their quality of life. Therefore, antifungals are used to treat this pathology [6,7].

Topical and oral therapies with antifungal agents are the most commonly employed treatments for onychomycosis. Many of these agents are synthetic and exhibit fungistatic action, such as azoles, tavaborole, terbinafine, amorolfine, and ciclopirox. However, both treatments have limitations. Oral therapy is limited by emerging antifungal resistance, potential hepatotoxicity, multiple side effects, and risk of drug interactions; in contrast, topical therapy is limited by the long treatment time and low drug permeation across the nail plate [2,8,9].

Therefore, research and development of new antifungal agents and new antifungal formulations are necessary to overcome the limitations of the available ones [10]. Alternative antifungal agents include bioactive compounds derived from plants, animals, microorganisms, nanotechnology processes, and antimicrobial combinations [11,12,13].

Essential oils are a potential alternative for the treatment of onychomycosis [14]. Essential oils are plant-derived lipophilic and volatile liquids composed mainly of terpenoids. They have pharmacological properties, including anti-inflammatory, antioxidant, analgesic, and antimicrobial activities, making them suitable for the pharmaceutical, healthcare, food, and cosmetic sectors [15,16]. Essential oils are generally recognized as safe (GRAS) for their intended use [17]. 

Oregano essential oil (OEO) has a broad spectrum of antimicrobial action, including against dermatophytes [18,19]. This oil is composed mainly of carvacrol and thymol, which are phenolic compounds directly responsible for the antimicrobial mechanism of OEO [20]. Carvacrol and thymol alter the permeability of the microbial cytoplasmic membrane and act as proton exchangers, expelling cytoplasmic content and acidifying the interior of the microorganism [21,22,23].

Rosemary essential oil (REO) also has an antifungal effect, including against fungi that cause onychomycosis [24]. This oil exhibits a polyphenolic profile containing carnosic acid, carnosol, rosmarinic acid, hesperidin, epirosmanol, rosmanol, 1,8-cineole, camphor, and α-pinene, among others. The composition of rosemary oil can vary between oils from different sources. The antimicrobial mechanism of REO occurs through the interaction of its terpenes and phenolic components with the microorganism’s cytoplasmic membrane, causing leakage of cytoplasmic content and morphological and functional alteration of the membrane [25]. 

Due to their composition and lipophilicity, both oils affect the lipid bilayer of the membrane, which is an essential structure for the microorganism [15]. Although the main components of both OEO and REO are directly responsible for their antimicrobial mechanism of action, their minor compounds also contribute indirectly to the bioactivity of oils, for example, by acting synergistically with the main active ingredients [26,27]. However, both essential oils present characteristic odors, which may limit their application [28]; therefore, combining them with another active ingredient is a strategy to minimize these undesirable organoleptic effects. Our research group proposes the association of OEO and REO with biogenic silver nanoparticles (bioAgNPs) to overcome these issues.

Nanotechnology has contributed to the development of antifungals, such as silver nanoparticles [29], which can be obtained by physical, chemical, or biological synthetic routes [30]. The advantage of green nanotechnology is that it leads to silver nanoparticle production using methods that are low-cost and less polluting than non-biological synthetic routes [31]. The present study utilized bioAgNPs, which were synthesized using green nanotechnology using the fungus *Fusarium oxysporum*. The synthesis of these bioAgNPs is well characterized and validated [21,32,33,34]. In addition, the antimicrobial activity, including against fungi, has been extensively studied by our research group [21,33,35,36].

Therefore, our research group proposes the development of nail lacquers containing OEO, REO, and bioAgNPs for onychomycosis treatment. This article reports the development and pharmacotechnical characterization of these nail lacquers and their antifungal efficacy tested using ex vivo assays and scanning electron microscopy. These nail lacquers may combat microbial resistance, as they have a combination of active ingredients that have been proven to minimize the emergence of resistance [21], in addition to positively impacting health and reducing social consequences in patients with onychomycosis.

The nail lacquer presented adheres to the 5-free marketing concept, indicating that the product is free of potentially allergenic components. This manuscript demonstrates that quantitative tests are the most suitable than agar diffusion techniques for investigating the antimicrobial efficacy of active ingredient candidates and formulations. 

## 2. Results

### 2.1. Analysis of the Active Ingredients

#### 2.1.1. Antifungal Efficacy Assessed by the Agar Diffusion Method

**Figure 1:**
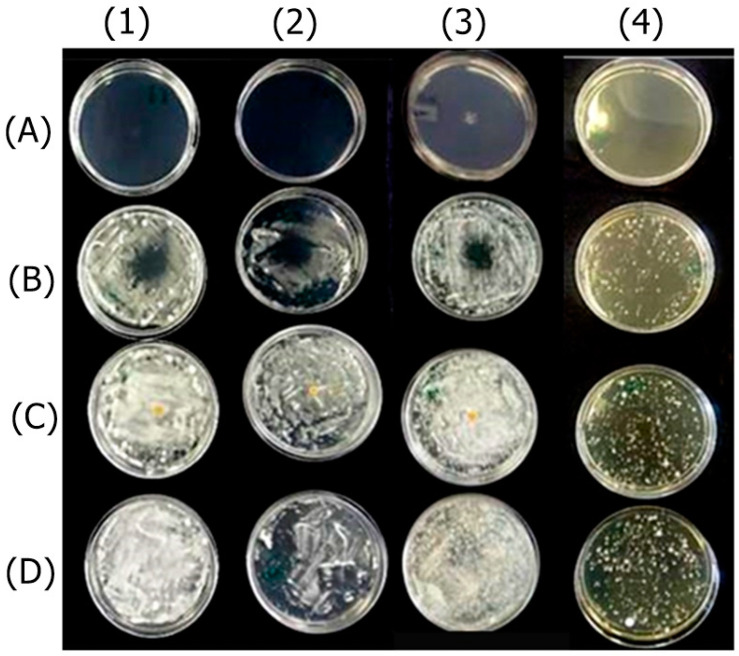
Antifungal effect of the active ingredients OEO (**A**), REO (**B**), and bioAgNPs (**C**) against the dermatophytes *Trichophyton mentagrophytes* (1), *Trichophyton rubrum* (2), *Microsporum canis* (3), and *Microsporum gypseum* (4). Cultures of the respective fungi, without the addition of active ingredients, were used as growth controls (**D**).

The OEO completely inhibited the fungal growth compared to the untreated control. Fungi treated with REO were not entirely inhibited but exhibited qualitatively reduced growth compared to the untreated control. The bioAgNPs demonstrated no antifungal effect, as evidenced by the agar diffusion technique, since fungi treated with bioAgNPs and the untreated control exhibited similar growth. The result is shown in Figure 1.

#### 2.1.2. Minimum Inhibitory and Minimum Fungicide Concentrations

All the active agents (OEO, REO, and bioAgNPs) inhibited the growth of all fungal species tested in this study (Table 1). All active ingredients are fungicides, as detailed in Table 2. The Supplementary Material includes Table S1, which shows the unit conversion for concentrations of essential oils and bioAgNPs.

#### 2.1.3. Cytotoxicity Evaluation

The CC_50_ values for each active compound against Vero cells are presented in Table 3.

### 2.2. Study of Nail Lacquer Formulations

#### 2.2.1. Antifungal Effect Using the Agar Diffusion Technique

**Figure 2:**
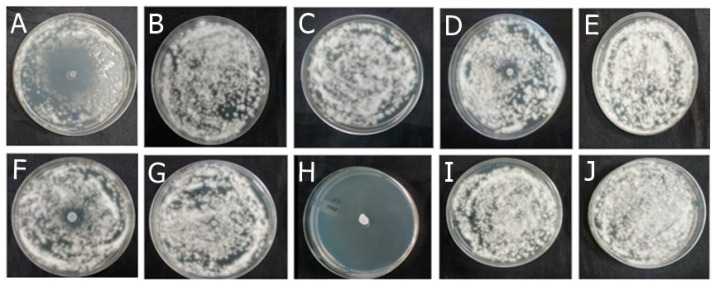
Antifungal effect of the formulations (F) with and without active ingredients (OEO, REO, and bioAgNPs), both individually and in combination, against *Trichophyton mentagrophytes*. The fungus was exposed to the formulations for 72 h. (**A**) F-OEO. (**B**) F-REO. (**C**) F-bioAgNPs. (**D**) F-OEO/REO. (**E**) F-REO/bioAgNPs. (**F**) F-OEO/bioAgNPs. (**G**) F-OEO/REO/bioAgNPs. (**H**) RF (reference formulation with amorolfine hydrochloride). (**I**) BF (base formulation without active). (**J**) Fungal growth control (without formulation).

**Figure 3:**
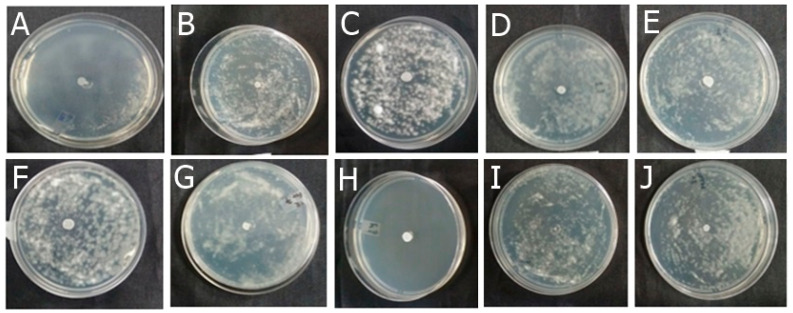
Antifungal effect of the formulations (F) with and without active ingredients (OEO, REO, and bioAgNPs), both individually and in combination, against *Trichophyton rubrum*. The fungus was exposed to the formulations for 72 h. (**A**) F-OEO. (**B**) F-REO. (**C**) F-bioAgNPs. (**D**) F-OEO/REO. (**E**) F-REO/bioAgNPs. (**F**) F-OEO/bioAgNPs. (**G**) F-OEO/REO/bioAgNPs. (**H**) RF (reference formulation with amorolfine hydrochloride). (**I**) BF (base formulation without active). (**J**) Fungal growth control (without formulation).

**Figure 4:**
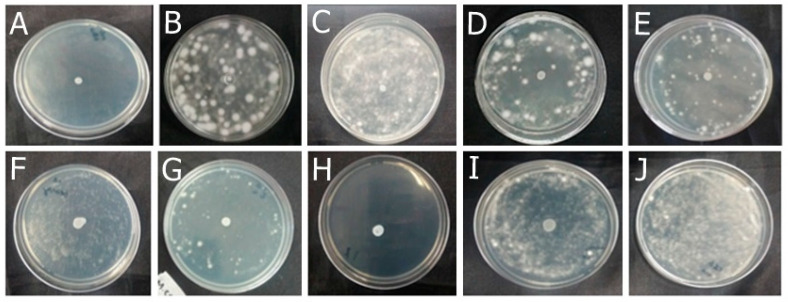
Antifungal effect of the formulations (F) with and without active ingredients (OEO, REO, and bioAgNPs), both individually and in combination, against *Microsporum canis*. The fungus was exposed to the formulations for 72 h. (**A**) F-OEO. (**B**) F-REO. (**C**) F-bioAgNPs. (**D**) F-OEO/REO. (**E**) F-REO/bioAgNPs. (**F**) F-OEO/bioAgNPs. (**G**) F-OEO/REO/bioAgNPs. (**H**) RF (reference formulation with amorolfine hydrochloride). (**I**) BF (base formulation without active). (**J**) Fungal growth control (without formulation).

**Figure 5:**
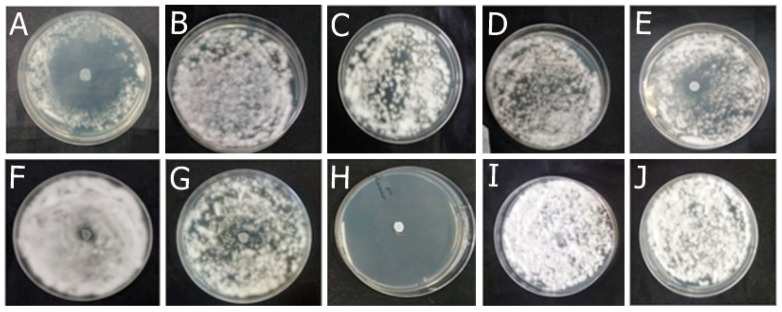
Antifungal effect of the formulations (F) with and without active ingredients (OEO, REO, and bioAgNPs), both individually and in combination, against *Microsporum gypseum*. The fungus was exposed to the formulations for 72 h. (**A**) F-OEO. (**B**) F-REO. (**C**) F-bioAgNPs. (**D**) F-OEO/REO. (**E**) F-REO/bioAgNPs. (**F**) F-OEO/bioAgNPs. (**G**) F-OEO/REO/bioAgNPs. (**H**) RF (reference formulation with amorolfine hydrochloride). (**I**) BF (base formulation without active). (**J**) Fungal growth control (without formulation).

Among the formulations developed in this study, F-OEO effectively inhibited the growth of all fungi tested. The fungal species tested include *Trichophyton mentagrophytes* (Figure 2), *Trichophytons rubrum* (Figure 3), *Microsporum canis* (Figure 4), and *Microsporum gypseum* (Figure 5), according to the agar diffusion technique. The BF did not exhibit any antifungal activity, indicating that the antifungal effect of F-OEO is due to the presence of its active ingredient.

#### 2.2.2. Ex Vivo Antifungal Effect

After 5 days of treatment, the formulations demonstrated antifungal efficacy. Only F-OEO, F-OEO/REO, F-OEO/bioAgNPs, and RF inhibited the growth of all fungi tested from the 5th to the 15th day of treatment, as shown in Table 4.

#### 2.2.3. Ex Vivo Permeation

**Figure 6:**
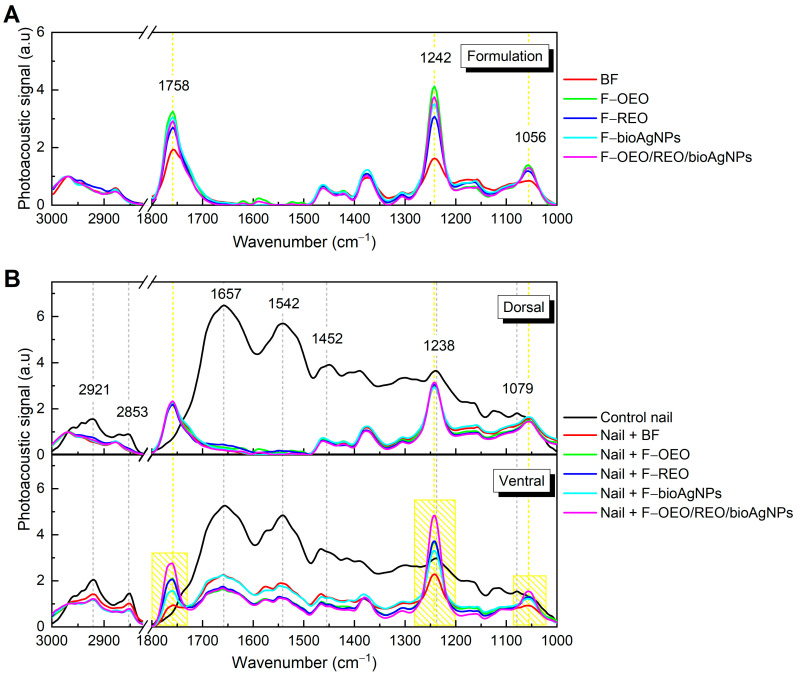
Absorption spectra obtained using FTIR-PAS of formulations and untreated and treated nails: (**A**) Formulations F-OEO, F-REO, F-bioAgNPs, F-OEO/REO/bioAgNPs, and BF. (**B**) Dorsal (top graph) and ventral (bottom graph) regions of the nail, both without nail lacquer (control) and after 30 min of contact with the formulations.

Figure 6A shows the FTIR-PAS absorption spectra (obtained in the region from 3000 to 1000 cm^−1^) of F-OEO, F-REO, F-bioAgNP, F-OEO/REO/bioAgNP, and BF; these formulations exhibited spectral patterns with variations in band intensity. The peaks centered at 1748, 1242, and 1056 cm^−1^ exhibited higher intensity in the spectra of formulations containing active ingredients compared to the blank formulation (BF) and were utilized as reference points in the permeation analysis. Figure 6B demonstrates the presence of these characteristic bands on the ventral surface of the nail after topical application of the formulations on its dorsal surface, indicating that permeation through the nail has occurred.

**Figure 7:**
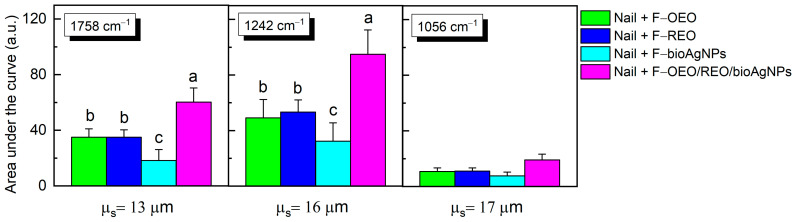
The area under the curve, obtained through integration for the bands centered at 1758, 1242, and 1056 cm^−1^, was calculated by subtracting the area of the control nail in this region. Each value represents the mean ± standard deviation of the group (n = 10 nails). The peaks centered at 1748, 1242, and 1056 cm^−1^ exhibited higher intensity in the spectra of formulations containing active ingredients compared to the blank formulation (BF) and were utilized as reference points in the permeation analysis. Statistical analysis was performed using one-way ANOVA followed by Tukey’s test with a significance level set at *p* < 0.05. Letters a–c indicate statistically significant differences (*p* < 0.05) among formulations in terms of permeation; different letters denote significant differences, whereas the same letters denote the absence of significant differences.

As shown in Figure 7, the mensurable permeation on the ventral surface of the nail was obtained by integrating the area under the absorption curve of formulations (band regions centered at 1758, 1242, and 1079 cm^−1^) subtracted from the area of the control nail. F-OEO/REO/bioAgNPs exhibit significantly greater permeation than all analyzed formulations; F-OEO and F-REO show similar permeation levels; and F-bioAgNPs exhibit significantly lower permeation than other formulations.

#### 2.2.4. Selection of the Best Nail Lacquer Formulations

F-OEO and F-OEO/bioAgNPs were chosen for further studies, including pharmacotechnical evaluation, stability analysis, and antifungal efficacy assessment via SEM.

#### 2.2.5. Pharmacotechnical Characterization of the Selected Formulations: Centrifugation Test, Organoleptic Characteristics, pH, Density, and Drying Time

The three selected formulations remained homogeneous after being subjected to centrifugation; their pre-stability renders them suitable for subsequent characterization. Both F-OEO and F-OEO/bioAgNPs formulations differed from BF in terms of color and odor. The F-OEO is slightly yellowish due to OEO, and the F-OEO/bioAgNPs is translucent light brown due to bioAgNP; both had a characteristic odor of OEO and maintained the same homogeneous appearance as the control. The formulations had a pH and density of approximately 4 and 10 g/cm^3^, respectively. The drying time for formulations ranged from 1 to 1.5 min. All the pharmacotechnical characteristics of formulations are shown in Table 5. The photodocumentation of the formulations is presented in the Supplementary Material (Figure S1).

#### 2.2.6. Preliminary Stability Study

Following exposure to thermal stress, the formulations F-OEO, FOEO/bioAgNP, and BF remained stable. They exhibited a homogeneous appearance without any signs of phase separation or precipitation. Additionally, they preserved their organoleptic and maintained a pH of 4.1, as presented in Table 5.

#### 2.2.7. Antifungal Efficacy Demonstrated by Scanning Electron Microscopy

**Figure 8:**
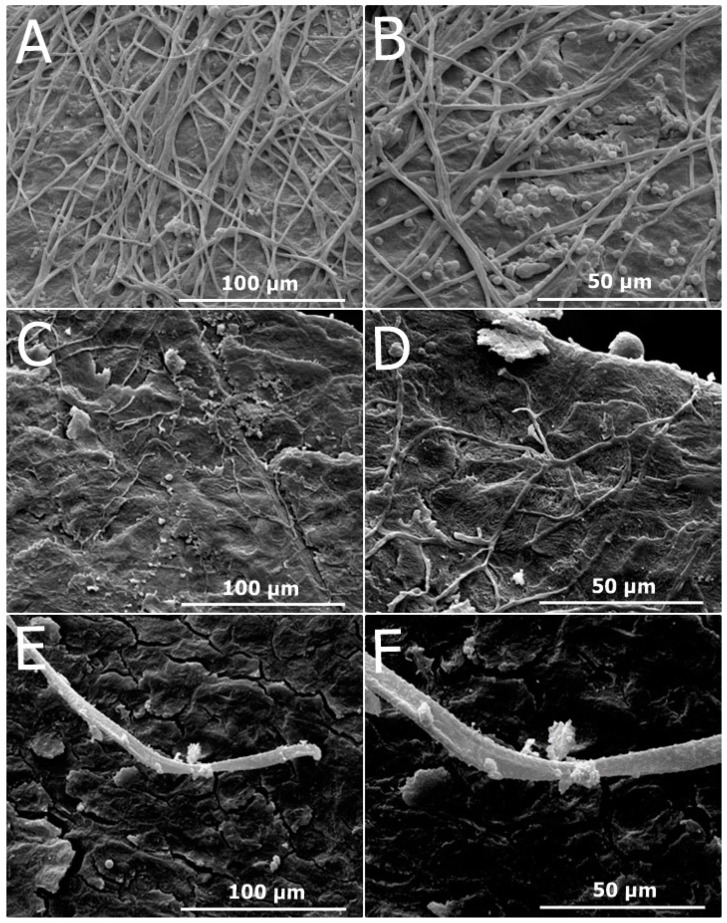
Scanning electron micrographs illustrating the antifungal effect of the formulations containing oregano essential oil (OEO) either alone or in combination with biogenic silver nanoparticles (bioAgNPs) against *Trichophyton mentagrophytes* grown on nails. (**A**) Untreated nail control (1200×). (**B**) Untreated nail control (2400×). (**C**) Nail treated with F-OEO (1200×). (**D**) Nail treated with F-OEO (2400×). (**E**) Nail treated with F-OEO/bioAgNPs (1200×). (**F**) Nail treated with F-OEO/bioAgNPs (2400×).

Figure 8A–C present scanning electron micrographs illustrating the effect of the formulations containing OEO (F-OEO and F-OEO/bioAgNP) against *Tricophyton mentagrophytes* grown in nails. The untreated nail sample exhibited hyphae with typical morphology (Figure 8A); a higher magnification micrograph reveals spores with typical size and hyphae and spores with intact surfaces (Figure 8B). Nail samples treated with F-OEO (Figure 8C,D) or F-OEO/bioAgNPs (Figure 8E,F) displayed an extremely reduced number of hyphae and spores compared to the untreated control. Fungal hyphae treated with F-OEO/bioAgNPs were damaged, appearing flatter than their typical morphology.

## 3. Discussion

Onychomycosis is a chronic fungal infection that is challenging to treat, as conventional therapies result in low cure rates, and antifungal resistance poses a global public health concern, exemplified by terbinafine-resistant dermatophytosis. Some conventional nail lacquers for topical treatment of onychomycosis exhibit low complete cure rates, ranging from 15.2% to 17.8% for amorolfine 5% (the reference formulation, RF, used in our study) [37]. Our study demonstrates the potent efficacy of new nail lacquers containing OEO and bioAgNPs as active ingredients, both individually and in combination, against dermatophyte fungi responsible for onychomycosis, such as *T. mentagrophytes*, *T. rubrum*, *M. canis*, and *M. gypseum*. Combination therapy with antifungals offers advantages over monotherapies, as it enhances product efficacy and reduces the risk of resistance emergence [38]. 

The agar diffusion technique demonstrated that OEO exhibits a pronounced and higher antifungal action compared to REO, and bioAgNPs do not inhibit fungal growth. Only formulations containing OEO, especially F-OEO, displayed antimicrobial effects using this technique. Although agar diffusion is a simple and low-cost test, it offers limited accuracy [39,40]. Due to its qualitative nature, this test did not reveal the antifungal effect of bioAgNPs and REO. The lack of an inhibition halo or the presence of only a small inhibition halo may be attributed to challenges associated with the diffusion of metal nanoparticles or essential oils in the agar due to their size [41] and lipophilicity [42], respectively. Furthermore, the formulations probably did not demonstrate an antifungal effect because the active agents are diluted in the nail lacquer vehicle, resulting in a concentration that is too low to diffuse effectively through the agar. Therefore, quantitative and more sophisticated tests were conducted to investigate the antifungal effect of the active ingredients and formulations presented in this study.

This research showed that the OEO has a fungicidal activity for all tested species. The three tested agents (OEO, REO, and bioAgNPs) inhibited the growth of the four dermatophyte species at low doses, consistent with previous studies. Our study showed MIC values lower than the data reported in the literature. As observed in our study, MIC values ranged from 0.05 to 0.09% *v*/*v* (0.47–0.85 mg/mL) for OEO, from 0.19 to 0.25% *v*/*v* (1.75–2.3 mg/mL) for REO, and from 2.48 to 6.23 µg/mL for bioAgNPs. Parrish et al. [21] reported that the MIC of OEO ranged from <0.12 to 0.5% *v*/*v* against *Trichophyton* spp. and from 0.5 to 0.25% *v*/*v* against *Microsporum* spp. Chaftar et al. [43] reported that REO MIC ranged from 1.80 to >8.80 mg/mL against various fungi, including dermatophytes. For silver nanoparticles with a diameter of 4 nm synthesized by photo-assisted reduction, Mousavi, Salari, and Hadizadeh [44] reported MIC values of 200 μg/mL against *M. canis*, 180 μg/mL against *T. mentagrophytes*, and 170 μg/mL against *M. gypseum*. For *F. oxysporym*-bioAgNP, the same nanosilver tested in our study, the MIC ranged from 4 to 8 μg/mL for *Aspergillus* species [33] and from 1.74 to 4.35 μg/mL for *Candida albicans* [45].

Some slight variations in MIC of compounds from different studies may occur due to variations in the source and composition of plant-derived or nanotechnological active ingredients. Additionally, different fungal strains used in various studies may have structural and metabolic variations that influence their sensitivity to the antimicrobials tested. Moreover, different studies employ distinct techniques for microbiological analysis, which affect the conclusion about antimicrobial activity.

OEO and REO are plant-derived agents, and their chemical composition can vary depending on climate, geography, and extraction methods [46,47]. Silver nanoparticles may vary in size, morphology, type and presence of stabilizing agents, and surface charge; these characteristics influence their antimicrobial activity [48]. The bioANPs tested in this study had an average diameter of 90 nm and a spherical shape, according to characterization conducted in a previous study [33]. In addition to size and morphology, other characterizations were previously performed for the bioAgNPs tested in the present study. The zeta potential of these nanoparticles is around −24.2 mV, and the polydispersity index (PDI) is around 0.357 [21]. Furthermore, we emphasize that these nanoparticles have already been characterized by X-ray diffraction (XRD), Fourier transform infrared spectroscopy (FTIR), and luminescence spectrophotometer, showing patterns of silver and characteristic bands of fungal proteins [32,33,34]. Additionally, bioAgNPs have fungal components, which may be important for their stabilization. The bioAgNPs are well studied and validated by our research group [21,32,33,35,49], demonstrating the importance of incorporating this well-characterized active into a nail lacquer for the treatment of onychomycosis. 

Despite the MIC variations discussed above, the antimicrobial properties of OEO, REO, and bioAgNPs are widely described in the literature. The antimicrobial effect of OEO is mainly due to its phenolic compounds, such as carvacrol and thymol [20,21,23]. The components of REO responsible for its antimicrobial activity are polyphenolic and terpene compounds such as carnosic acid, carnosol, rosmarinic acid, hesperidin, 1,8 cineole, camphor, α-pinene, and β-pinene, among others [25]. For silver nanoparticles, there is evidence that the antimicrobial activity is influenced by the release of Ag^+^ ions, but it is important to consider that the nanoparticle coat can influence its mechanism of action, and the coat varies for nanoparticles from different studies [48,50]. The composition of the active ingredient influences its antifungal activity as well as its toxicity, which is why a preliminary cytotoxicity test was conducted in our research.

According to the results of the present study, the OEO exhibited low toxicity to Vero cells since their CC_50_ (>7% *v*/*v* or >66 mg/mL) was greater than its MIC range (0.05–0.09% *v*/*v*). The CC_50_ of REO and bioAgNPs were 0.05% *v*/*v* (1.46 mg/mL) and 2.26 mg/mL, respectively. Ribeiro et al. reported that the percentage of remaining living keratinocyte cells was 85.95%, 82.16%, 4.28%, and 3.74% for treatment with 16 μg/mL, 32 μg/mL, 125 μg/mL, and 250 μg/mL of OEO, respectively [51]. Huang et al. reported that the CC_50_ range of six REO on keratinocytes was 1.095–2.549 mg/mL, as each oil varies in chemical composition [52]. Chen et al. study demonstrated that bioAgNPs are more toxic to keratinocyte monolayers than in the three-dimensional keratinocyte model; the same concentration of bioAgNPs reduced the viability of the monolayer by approximately 50% and by 10% in the three-dimensional model [53].

Vero cells were chosen for our study because they are mentioned in ISO 10993-5 [54] as a standard for biocompatibility testing. In addition, Vero cells are well-established and standardized, making it easier to compare results between different studies. They exhibit consistent growth under controlled conditions, improving the reproducibility of experiments and results. Their cultivation is relatively easy because they do not require elaborate or expensive culture media, making them more accessible for routine laboratory work [55].

Keratinocyte is the ideal cell for testing topical formulations. However, they present significant challenges in laboratory work. This cell line requires specific culture conditions that mimic the skin environment, making the cultivation complex and more time-consuming in routine laboratory procedures. These cells are highly sensitive to environmental conditions, such as pH, temperature, and humidity, which can influence test outcomes. Keratinocytes can exist in various states of differentiation, ranging from basal to fully differentiated cells, which may affect their response to different substances and complicate the interpretation of results [56,57]. The monolayer cell culture model, although commonly used, is extremely sensitive. As demonstrated by the study of Chen et al. [53], this monolayer cell model does not accurately represent the skin barrier or its physiological responses.

Some studies test the cytotoxicity of ingredients individually rather than the formulation as a whole due to the challenges posed by testing the entire formulation in conjunction with cells. The formulations can present difficulties in being added to cell cultures, and the test may require adaptations. Testing the toxicity of formulations with an in vitro cell model is challenging because, in addition to the active ingredient, these products contain alcohol-based excipients or surfactants. For example, isopropyl alcohol serves as a lipid solubilizer and affects biological membranes [58].

Some studies test the cytotoxicity of formulations by traditional methodologies involving products without solvents or surfactants that may have antimicrobial action, such as hydrogel pharmaceutical forms. Some studies also test concentrations of the formulation, which often results in the dilution of active ingredients and excipients, thereby not representing the correct way to use the product in its undiluted form. Alternatively, they adapt the test conditions so that the product does not undergo dilution. Urzedo et al. [59] reported that the cytotoxicity of the hydrogel at 2 μg/mL containing bioAgNPs was not toxic to Vero cells, with more than 70% cell viability observed. Similarly, Sruthi et al. [60] demonstrated that hydrogel containing the plant-derived enzyme papain had a CC_50_ of 130.5 μg/mL for Vero cells. Limmatvapirat et al. [61] reported that a nail lacquer did not exhibit cytotoxicity to fibroblasts. Valdes et al. [62] reported that polyurethane nail lacquers for the delivery of terbinafine showed good keratinocyte compatibility, with all samples treated with the formulations demonstrating 70% cell viability.

OEO and bioAgNPs were selected for incorporation into the final nail lacquer formulation. OEO exhibits potent antifungal activity and is non-toxic at the concentration used in the nail lacquer. BioAgNPs were incorporated into the formulations at a concentration of 1.25% *v*/*v* (2.12 µg/mL), which is effective in providing an antifungal effect but is near its CC_50_ value. Samberg et al. [63] reported that the toxicity of silver nanoparticles may be influenced by the vehicle of the colloidal suspension; thus, the purification and washing steps of bioAgNPs may reduce their cytotoxicity.

OEO and REO are considered GRAS by the Food and Drug Administration (FDA) [17], which justifies our choice to incorporate them as active ingredients into nail lacquer formulations. Our study also developed formulations containing OEO and REO in combination with bioAgNPs; this combined antimicrobial therapy was employed as a strategy to reduce the required dose of each active and to combat antifungal resistance [21,35,49,64,65].

The ex vivo nail infection assay was selected to evaluate the antifungal efficacy of the nail lacquer, as it simulates the conditions of an onychomycosis treatment with the application of formulations directly to previously infected nails. Only F-OEO, F-OEO/REO, and F-OEO/bioAgNPs demonstrated antifungal efficacy comparable to the commercial formulation containing amorolfine hydrochloride; these formulations inhibited the growth of the four fungal species after five days of treatment. Among the available antifungal therapies for onychomycosis, amorolfine is notable for its clinical use and was, therefore, chosen as RF. Other antifungals on the market for onychomycosis include ciclopirox, efinaconazole, and tavaborole. Amorolfine and ciclopirox require application once or twice a day for 24 to 48 weeks. Eficonazole and tavaborole have low permeation and long treatment times, with low cure rates (7 to 29%) [2,8,9,66]. The formulations developed in our study have a quick antifungal effect (within 5 days), with treatment administered every other day; in addition, they demonstrate good permeation into the nail.

Valdes et al. [62] reported the development of a nail lacquer containing 1% (*w*/*w*) terbinafine, which exhibited activity against *T. rubrum* and other fungal species. Yadav, Mishra, and Vishwakarma [67] reported that an antifungal nail lacquer containing miconazole nitrate hydrochloride had an effect on *Candida albicans*. Nikhath and Sanjana [68] found that lacquer with oxiconazole had an effect on *C. albicans*. Although the literature includes studies on the antifungal efficacy of various nail lacquers, the tests employed different techniques and qualitative methods, making comparisons with our results very difficult. Therefore, we emphasize the necessity of standardizing quantitative tests to enable the comparison of antifungal efficacy results across different studies.

To promote the treatment of onychomycosis, the antifungal nail lacquer must effectively permeate the nail. The FTIR-PAS absorption spectra of formulations (F-OEO, F-REO, F-bioAgNPs, F-OEO/REO/bioAgNPs, and BF) before application on the nail exhibited similar spectral patterns with variations in band intensity. The principal spectral peaks identified are attributed to C-H stretching of hydrocarbons at 2968 and 2877 cm^−1^, C=O stretching in carbonyl groups at 1758 cm^−1^, C-O bonds at 1242 cm^−1^, and C-O-C bonds at 1056 cm^−1^ [69,70,71]. The control nail presented peaks centered at 2921 and 2853 cm^−1^, which are attributed to CH_2_ stretching of lipids; at 1657, 1542, 1452, and 1238 cm^−1^, corresponding to bands I, II, and III of the amide functional group; and at 1079 cm^−1^ of the C-C bond of the DNA backbone [72]. After 30 min of contact between the nail and the formulation, the spectra show an increase in peak intensity at 1758, 1242, and 1079 cm^−1^, indicating absorption of the nail lacquer. The presence of these bands on the ventral surface of the nail demonstrates the permeation capability of the nail lacquer.

The permeation of an active ingredient through the nail plate may be influenced by its physicochemical properties (such as size, charge, and lipophilicity), the formulation characteristics (e.g., vehicle nature, pH, concentration of the active ingredient), the state of the nail (e.g., degree of hydration and stage of the disease), and interactions with the keratin network present in the nail [1,73]. 

The molecular weight of an antifungal agent is a critical property influencing its ability to permeate the nail plate; the higher the molecular weight, the lower the extent of permeation. The increase in lipophilicity is concomitant with the increase in molecular weight of compounds of the same class (e.g., n-alcohols), resulting in reduced permeation. Essential oils are composed of molecules with low molecular weight molecules; therefore, their permeation through the nail plate is facilitated [73,74].

The charge or absence of charge of an active substance is also a relevant parameter for its permeation. Non-ionic agents can be up to 10 times more permeable than their ionized counterparts. In this context, the low permeability of the ionized agent is possibly due to two factors: (1) the increase in the size of the structure caused by the hydration of the ionized species and (2) the electrostatic repulsion between the charges of the species and the keratin, if they are similar [73]. In our study, the bioAgNPs release Ag^+^ ions that may undergo hydration; this likely explains the low permeation of the F-bioAgNPs formulation.

Nails affected by onychomycosis tend to become thicker and more porous, which increases the permeation of the active ingredient from the dorsal to the ventral region. The degree of hydration of the nail also seems to facilitate nail permeation [1]. It is important to highlight that permeation can be improved by the use of physical methods, such as sanding, lasers, and photodynamic therapy, or keratolytic agents, such as urea, thioglycolic acid, and salicylic acid [75,76]. Additionally, a permeation enhancer can be incorporated into the formulation of nail lacquer.

Based on antifungal efficacy and permeation data, the formulations F-OEO and F-OEO/bioAgNPs were selected for pharmacotechnical analysis and antifungal confirmation via SEM. All the formulations (BF, F-OEO, and F-OEO/bioAgNPs) exhibited stability without any macroscopic alteration after being subjected to the 3200 rpm. It is important to note that centrifugation simulates the effect of gravity on the sample. Therefore, this test allows us to predict potential instabilities that may arise in the product in the future, such as sedimentation, phase separation, or coalescence [77]. Since all formulations passed the centrifugation test, they were subsequently subjected to thermal stress in a second stability test (preliminary stability) [78].

After being subjected to thermal stress, the formulations remained stable with respect to their pH and organoleptic characteristics. BF, F-OEO, and F-OEO/bioAgNPs formulations maintained their acidic pH (4–4.4), which is consistent with the pH of the nail [79]. The pH of the nail is reported in the literature to be between 4 and 6, with an average value of 5. This acid pH is important for defense against microorganisms. Toenails exhibit a less acidic pH compared to fingernails, which promotes the production of spores by *Trichophyton rubrum*, one of the most common fungal causes of onychomycosis [79,80]. Our formulations are very close to the typical pH range of commercial nail lacquers (approximately 4.5 to 5.5), ensuring compatibility with the natural pH of the nail and skin. If an adjustment is necessary to increase the pH slightly, the formulation can be alkalized to achieve the desired pH. All three formulations maintained a homogeneous liquid aspect. Their color and odor remained unchanged; F-OEO retained a slightly yellowish color, F-OEO/bioAgNPs appeared as translucent light brown, and BF remained colorless. The formulation containing OEO preserved its characteristic odor of OEO oil.

According to Joshi, Sharma, and Pathak [81], the time required for nail lacquer to form a film ranges from one to two minutes. The formulations BF, F-OEO, and F-OEO/bioAgNPs exhibited drying times shorter than two minutes within this reference range. The F-OEO formulation dried more quickly than the BF; the presence of OEO reduced the drying time, as essential oils are easily volatilized [16]. The F-OEO/bioAgNP formulation took the longest time to dry; the presence of the bioAgNPs likely delayed solvent evaporation.

The SEM observations confirmed the antifungal effect of nail lacquers F-OEO and F-OEO/bioAgNPs on contaminated nails, which was previously detected in the ex vivo assay presented in this manuscript. This analysis by electron microscopy revealed an intense reduction in the number of hyphae and the absence of spores in treated nails compared to untreated nails (control). Morphological alterations in hyphae (such as flat hyphae) were observed in the F-OEO/bioAgNP-treated sample, suggesting the extravasation of intracellular content. The topography of untreated nails was obscured by fungal structures covering the nail surface, which corroborates with the micrographs of infected nails reported by Lana et al. [82]. In contrast, the treated nails, which were free of fungi structures (Figure 8E), exhibited preserved nail morphology with clearly visible and organized keratin layers; these observations are consistent with findings from other studies [82,83].

Studies have demonstrated that OEO, carvacrol, thymol, and nanosilver increase the membrane permeability of microorganisms, leading to the loss of their cytoplasmic contents [21,22,23,48,50]. Utilizing SEM analysis, Zulu et al. [84] observed that OEO caused alterations in hypha morphology, such as reduced volume and the occurrence of breakage in *Penicillium digitatum*. Bocate et al. [33] reported that exposure of *Aspergillus ochraceus* to bioAgNPs strongly reduced spore germination and induced fungal cell damage, resulting in the formation of short and unbranched hyphae.

This study presents two nail lacquer formulations, F-OEO and F-OEO/bioAgNPs, as promising alternatives for the topical treatment of onychomycosis. The combination of OEO and bioAgNPs in the nail lacquer is strategic to both improve their antimicrobial activity and combat microbial resistance. A previous study conducted by our research group on the antimicrobial mechanism of this combination suggests that the oil increases the permeability of the microorganism cytoplasmic membrane, thereby facilitating the entry of nanosilver. Furthermore, this combination prevented the emergence of resistance to both antimicrobials in a microorganism test model [21]. This is likely because the combination inhibits the activation of the metal resistance mechanism, which typically occurs via an efflux pump [85], since the combination, primarily due to the oil, targets the cytoplasmic membrane.

This manuscript reports the preclinical phase of the development of products intended for the topical treatment of onychomycosis. The products developed in this study present a technological maturity level of 5, as they have been tested and validated in vitro and ex vivo [86]. Further studies are needed to characterize these formulations in detail from a physicochemical perspective and to investigate the efficacy of the formulations in the treatment of onychomycosis. A clinical trial will be conducted with both healthy and sick individuals to integrate all the necessary elements for these antifungal nail lacquers to be used safely and effectively by patients with onychomycosis.

## 4. Materials and Methods

### 4.1. Antifungal Agents

#### 4.1.1. Essential Oils

OEO and REO were obtained from Ferquima Industry and Commerce of Essential Oils (São Paulo, Brazil). Both oils were extracted from leaves via steam distillation, and their main characteristics were detailed in a technical report provided by the company. The density was 0.9468 g/mL for OEO and 0.9130 g/mL for REO. The primary components of OEO were as follows: 72% carvacrol, 2% thymol, 4.5% gamma-terpinene, 4% para-cymene, and 4% linalool. The main components of REO were as follows: 40% 1,8-cineole, 15% camphor, 13% alpha-pinene, 7% beta-pinene, and 3% limonene.

#### 4.1.2. Biogenically Synthetized Silver Nanoparticles (bioAgNPs)

The bioAgNPs were produced in the Department of Microbiology at Universidade Estadual de Londrina. They were synthesized biogenically according to a previously established method using *F. oxysporum* [32]. The fungus was cultivated in a liquid medium containing malt extract (2%) and yeast extract (5%) for 7 days at 30 °C. The fungal biomass was collected by filtration and added at a concentration of 0.1 g/mL in sterile distilled water. This suspension was kept under agitation (150 rpm) for 72 h at 30 °C. Subsequently, the aqueous solution components were separated by filtration. AgNO_3_ at 1 mM (170 µg/mL) was added to the solution, which was then incubated at 30 °C in the dark for 14 days. Following the electrochemical reduction of silver nitrate by fungal-free solution components, the bioAgNPs were obtained and characterized. Their size was analyzed using a Zeta-APS instrument (Matec Applied Sciences, USA), and their morphology was examined using scanning electron microscopy.

### 4.2. Development of Nail Lacquer Formulations

**Figure 9:**
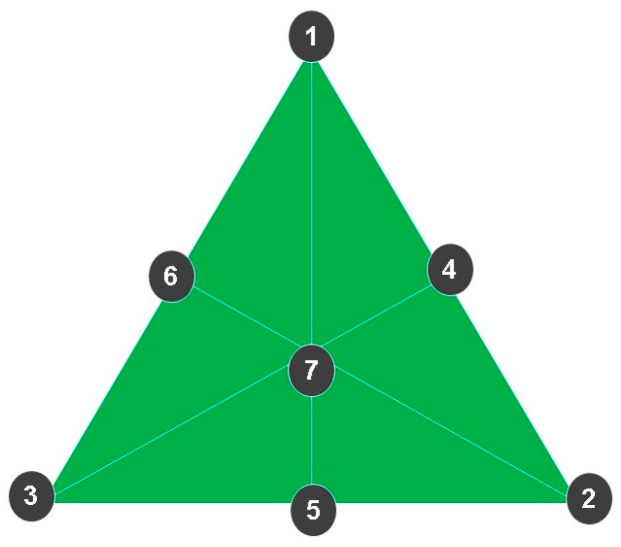
The simplex-centroid experimental design was used to define the concentration of actives both alone and in binary or ternary combinations. (1) Formulation containing only OEO. (2) Formulation containing only REO. (3) Formulation containing only bioAgNPs. (4) Formulation containing OEO and REO. (5) Formulation containing REO and bioAgNPs. (6) Formulation containing OEO and bioAgNPs. (7) Formulation containing the ternary combination of actives: OEO, REO, and bioAgNPs.

The nail lacquer formulation was developed based on the 5-free marketing concept, which means that the products are free of potentially allergenic components such as dibutyl phthalate (DBP), 2-nitrotoluene, toluene, furfural, and formaldehyde. All formulations were composed of the following ingredients: cellulose acetate butyrate (Eastman), isopropyl alcohol (Labsynth), ethyl acetate (Labsynth), and sucrose acetate butyrate (Eastman). The simplex-centroid experimental design was used to optimize the concentrations of active ingredients alone and in binary or ternary combinations [87], as shown in Figure 9 and and Table 6.

A commercial nail lacquer containing 5% (*w*/*v*) of amorolfine hydrochloride was used in this study as a reference formulation (RF) for comparative purposes.

### 4.3. Pharmacotechnical Characterization of Nail Lacquer Formulations

The pharmacotechnical evaluation of the formulations was carried out to ensure their quality and efficacy. Several tests were performed, including a pre-stability study, organoleptic and physicochemical characterization, drying-time determination, and a preliminary stability study.

#### 4.3.1. Pre-Stability Study 

For the pre-stability study, 5 g of each formulation was placed in conical test tubes and subjected to centrifugation at 3200 rpm for 30 min at 25 °C. Subsequently, each formulation sample was visually analyzed to detect possible signs of instability, such as coalescence, phase separation, and sedimentation [78].

#### 4.3.2. Organoleptic Characterization

The formulations were evaluated for their appearance, color, and odor in the transparent glass bottle in which they were packaged after 24 h of their production, according to the methodological guide [77]. For appearance analysis, the formulations were visually inspected to detect the presence or absence of alterations such as phase separation, precipitation, or turbidity. The color of each formulation was visually assessed under white light against a black background. Their odor was directly evaluated by human olfactory perception.

#### 4.3.3. Physicochemical Characterization

The pH value of each formulation was determined using a digital pH meter calibrated with buffer solutions (pH 4 and pH 7) at 25 ± 5 °C, with the electrode inserted directly into the sample [84].

The evaluation of density was carried out using a glass pycnometer. The density values of formulations were obtained using Equation (1) [77].

(1) $$d=\left(\frac{M_{2}-M_{0}}{M_{1}-M_{0}}\right)$$

where

d = Relative density of formulation in g/cm^3^

M_2_ = Mass in g of the pycnometer containing the formulation

M_1_ = Mass in g of the pycnometer containing water

M_0_ = Mass in g of the empty pycnometer

#### 4.3.4. Determination of Film Drying Time

The drying time of the developed nail lacquers was evaluated by applying a thin layer of formulation (100 µL) onto a Petri dish using an applicator, according to Shah and Jobanputra [88], with modifications. The time required for the nail lacquer to form a dry and touchable film was determined. This test was conducted in triplicate, and the result was expressed in seconds, representing the average of the three measurements.

#### 4.3.5. Preliminary Stability Study

The preliminary stability study was conducted in accordance with the Cosmetic Products Stability Guide [78]. The formulations were subjected to thermal stress for a duration of 15 days, following a 24 h cycle of altering temperature: 4 ± 2 °C or 40 ± 2 °C (75 ± 5% RH). After 15 days, the formulations were evaluated for pH and organoleptic parameters. The results obtained were compared with those observed prior to the stability test.

### 4.4. Antifungal Activity of Actives and Nail Lacquer Formulations

#### 4.4.1. Fungal Species

Four fungal species were utilized in this study: *T. rubrum*, *T. mentagrophytes*, *M. canis*, and *M. gypseum*. The microorganisms were provided by the Laboratory of Medical Mycology and Oral Microbiology of Universidade Estadual de Londrina (Londrina, Paraná, Brazil). All fungal samples were stored in a solution containing 40% (*v*/*v*) glycerol (Merck, Darmstadt, Germany) at −20 °C.

To prepare fungal cultures for antimicrobial efficacy testing, each microorganism was initially transferred to test tubes containing potato dextrose agar (BDA) and incubated at 25 ± 5 °C for approximately 14 days.

#### 4.4.2. Analysis of Antifungal Activity of Actives and Formulations by the Disk Diffusion Method

This assay was conducted in triplicate, according to Nweze et al. [89], with modifications. A volume of 100 µL of fungal spore suspension at 10^4^/mL was spread over the surface of the BDA in a Petri dish. Subsequently, 10 µL of the active ingredient (OEO, REO, or bioAgNPs) or formulations was applied to the center of the agar surface in the Petri dish. The fungus inoculated with the test compound was incubated at 25 ± 5 °C for 72 h. The antimicrobial effect of the active ingredients or formulations was assessed based on the absence or reduction in mycelial fungal growth. 

#### 4.4.3. Determination of Minimum Inhibitory Concentration (MIC) of the Actives

The MIC for each antimicrobial agent (OEO, REO, and bioAgNP) was conducted using broth microdilution [90,91]. For OEO and REO, an alcoholic solution was initially prepared at a concentration of 40% (*v*/*v*) for each essential oil. The bioAgNPs were not diluted in alcohol as they are colloidal aqueous suspensions. Tested concentration ranges were as follows: 0.0005–1% (*v*/*v*) for OEO, 0.0005–1% (*v*/*v*) for REO, and 0.02–42.5 µg/mL for bioAgNP. The concentrations were prepared by serial dilution in RPMI medium. RPMI alone and RPMI containing each of the antimicrobials separately were tested as sterility controls. Untreated fungi inoculated into RPMI were used as growth controls. The MIC was defined as the lowest antimicrobial concentration that inhibited visible growth after 72 h of treatment at 25 ± 5 °C. All assays were performed in triplicate.

#### 4.4.4. Determination of Minimum Fungicidal Concentration (MFC) of the Actives

The MFC of each antimicrobial (OEO, REO, or bioAgNP) was determined by subculturing 10 µL of fungal culture treated with MIC and higher concentrations into a BDA medium without antimicrobials. After incubation at 25 ± 5 °C for 72 h, the MFC was defined as the lowest concentration required to completely eradicate the tested fungus [92]. All assays were performed in triplicate.

#### 4.4.5. Ex Vivo Evaluation of Antifungal Efficacy of the Nail Lacquer Formulations

Human nail samples were collected from both female and male volunteers aged 18 to 60 years. The nails were obtained through standard cutting techniques without the need for specific training for the task; they were stored in a clean container and then autoclaved for later use. As cut nails are considered ex vivo material, there was no need to submit this study to the ethics committee.

Ex vivo evaluation of the antifungal efficacy of the nail lacquer formulations was performed according to previous studies [82,93], with modifications. Nail units were immersed in 10 mL of fungal spore suspension at 10^5^/mL. The fungal suspension containing nails was incubated at 25 °C with agitation at 150 rpm for 2 h. Subsequently, the contaminated nails were transferred to Petri dishes containing cotton moistened with sterilized water; they were incubated at 25 ± 5 °C for 14 days. After this period, the nails were treated with the formulations, including BF and RF, on alternate days for 15 days. On the 5th, 10th, and 15th days of treatment, a nail fragment was excised and transferred to PDA medium to evaluate microbial growth.

#### 4.4.6. Investigation of Antifungal Lacquer Efficacy by Scanning Electron Microscopy

Nail fragments infected with *T. mentagrophytes* and post-treated with the selected formulations were analyzed by scanning electron microscopy (SEM). Untreated nails served as the control. For SEM analyses, sample (both treated and untreated nails) preparation was performed according to Oliveira et al. [94], with modifications. Each nail sample was fixed by immersion in 0.1 M sodium cacodylate buffer (pH 7.2) containing 3% (*v*/*v*) formaldehyde and 2% (*v*/*v*) paraformaldehyde for 1 h, followed by three washing steps (10 min each) with sodium cacodylate. Then, samples were post-fixed in OsO_4_ 1% for 1 h at room temperature and were washed again as previously described. Fixed nails were dehydrated in an ethanol gradient (50%, 60%, 70%, 80%, 90%, and 100% *v*/*v*), critical point-dried using CO_2_ (BALTEC CPD 030 Critical Point Dryer), coated with gold (BALTEC SDC 050 Sputter Coater), and observed under a SEM (FEI Quanta 200).

### 4.5. Analysis of Nail Lacquer Permeation

The permeation study of formulations was accessed by Fourier transform infrared photoacoustic spectroscopy (FTIR-PAS). Prior to the measurements, the photoacoustic cell and sample were purged with helium gas, and the cell was then sealed. The spectra were collected in the spectral range between 3000 and 1000 cm^−1^, with a resolution of 8 cm^−1^ and an average of 128 scans. Photoacoustic spectra were obtained by initially illuminating the dorsal side of the nail, followed by the ventral side. The depth of the nail sample contributing to the photoacoustic signal was estimated using the thermal diffusion length (µ_s_) (Equation (2)), taking the thermal diffusivity of the nail as D = 10 × 10^−4^ cm^2^/s [95] and the modulation frequency of f = 303.74 at 101.25 Hz. The value of µ_s_ ranges from 10 to 18 µm.

(2) $$\mu_{s}={\left(\frac{D}{\pi f}\right)}^{1/2}\;;\;\mathrm{with}\;f=\frac{\nu \times f\prime }{\nu \prime }$$

where

D = Thermal diffusivity of the sample (cm^2^/s)

f = Modulation frequency at the chosen wavenumber (Hz)

f’ = Laser modulation frequency

v = Wavenumber

v’ = Laser wavenumber

**Figure 10:**
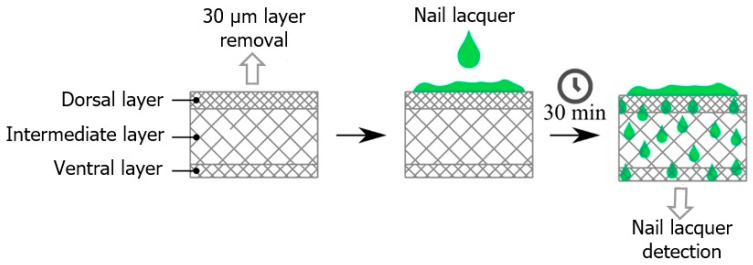
Schematic diagram illustrating the permeation of nail lacquers BF, F-OEO, F-REO, F-bioAgNPs, and F-OEO/REO/bioAgNPs through the nail. This ex vivo assay involved the application of the formulations to the dorsal surface of the nails. After 30 min, the ventral surface was analyzed using FTIR-PAS.

Nail fragments were obtained from an adult volunteer aged 23 years old. The nail samples were manually cut into similar parts with approximately 0.3 × 0.3 cm and an average thickness of 408 µm. Before applying the formulation, approximately 30 µm was removed from the dorsal surface of the nail using sandpaper. Subsequently, the FTIR-PAS spectrum of both the dorsal and ventral nails was recorded; spectra of the nail lacquer formulations alone were also obtained. Then, 5 µL of the formulation was applied to the dorsal surface of the nail. After 30 min, spectral measurements were taken of the nail to evaluate the permeation of the formulation. Sample preparation is illustrated in Figure 10.

### 4.6. Cytotoxicity Assay of Antifungal Actives

Toxicity analysis of the active ingredients was conducted using Vero cell lines (monkey kidney cells) in 96-well plates. Vero cells were cultured in RPMI medium 1640 (Gibco) at 37 °C with 5% CO_2_ until a monolayer was established in the wells. Non-adherent cells were removed by washing with PBS. Then, confluent cells were treated for 24 h at 37 °C in 5% CO_2_ with various concentrations of active ingredients, with the maximum concentrations being 7% (*v*/*v*) for OEO, 7% (*v*/*v*) for OEA, and 4.25 µg/mL for bioAgNP. After the treatment period, the medium was removed, and the wells were gently washed with PBS. Cell viability was assessed using the MTT [3-(4,5-dimethyl-2-thiazolyl)-2,5-diphenyl-2H-tetrazolium] assay, following the manufacturer’s instructions. Untreated Vero cells were used as the control, representing 100% viability. The 50% cytotoxic concentration (CC_50_) was defined as the concentration required to reduce cell viability by 50% compared to the untreated control.

### 4.7. Statistical Analysis

The data were subjected to analysis of variance (ANOVA), followed by the Tukey test. The confidence interval was set at 95%.

### 4.8. Experimental Design

**Figure 11:**
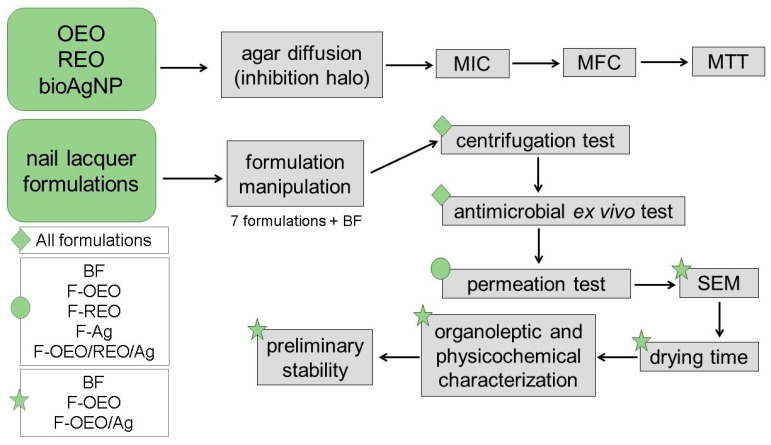
Schematic diagram of the experimental procedure. Initially, the active ingredients (OEO, REO, and bioAgNPs) were evaluated against dermatophyte fungi (*T. rubrum*, *T. mentagrophytes*, *M. canis*, and *M. gypseum*) using agar diffusion and broth microdilution techniques to determine the fungal growth inhibition halo and the efficient concentrations (MIC and MFC) of each active, respectively. The cytotoxicity of actives was assessed using the MTT assay. Then, the nail lacquer formulations were prepared and subjected to the following tests in sequence: stability centrifugation test, antimicrobial ex vivo test using fungal-contaminated nail (performed against the four fungal species), permeation analysis across the nail using FTIR-PAS, an antifungal study by SEM, drying time assay, evaluation of organoleptic and physical–chemical characteristics, and preliminary stability study. The tests were performed in the order indicated by the arrows. Some tests were performed with all formulations, while others were carried out with some formulations. Green diamond: indicates all formulations. Green circle: indicates five formulations. Green star: indicates the promising formulations for the final product.

Figure 11 illustrates the experimental design of this study, outlining the methodological steps in the sequence in which they were conducted.

## 5. Conclusions

In conclusion, F-OEO and F-OEO/bioAgNPs represent promising antifungal alternatives for the topical treatment of onychomycosis. Both formulations exhibited potent antifungal efficacy, effective permeation through the nail, proven stability, and physical–chemical properties appropriate for nail lacquer. F-OEO and F-OEO/bioAgNPs demonstrated an antifungal effect comparable to RF (reference formulation, which contains amorolfine hydrochloride as the active ingredient). However, the nail lacquers we developed incorporate active agents derived from green nanotechnology and plant-based ingredients classified as GRAS by the FDA. Both formulations also adhere to the 5-free marketing concept, indicating that the product is free of potentially allergenic components. Further studies are required to evaluate the clinical efficacy of these nail lacquers in treating onychomycosis. The present research also highlights that quantitative tests, such as the determination of MIC and MFC, are more suitable than qualitative tests, such as agar diffusion, for assessing the antifungal efficacy of active agents. The ex vivo test, which involves prior infection and subsequent nail treatment, constitutes an appropriate model for assessing the antifungal efficacy of nail lacquer, as it closely mimics the conditions of onychomycosis.

## Figures and Tables

**Table 1:** Mean minimal inhibitory concentrations of oregano essential oil (OEO), rosemary essential oil (REO), and biogenic silver nanoparticles (bioAgNPs).

Fungal Species	OEO (%, *v*/*v*)	REO (%, *v*/*v*)	bioAgNPs (µg/mL)
*Trichophyton mentagrophytes*	0.05 ± 0.02	0.19 ± 0.09	4.98 ± 1.70
*Trichophyton rubrum*	0.05 ± 0.02	0.19 ± 0.09	2.48 ± 0.00
*Microsporum canis*	0.09 ± 0.04	0.19 ± 0.09	3.28 ± 0.00
*Microsporum gypseum*	0.06 ± 0.00	0.25 ± 0.00	6.23 ± 0.00

**Table 2:** Minimum fungicidal concentrations of oregano essential oil (OEO), rosemary essential oil (REO), and biogenic silver nanoparticles (bioAgNPs).

Fungal Species	OEO (%, *v*/*v*)	REO (%, *v*/*v*)	bioAgNPs (µg/mL)
*Trichophyton mentagrophytes*	0.13	0.25	>42.50
*Trichophyton rubrum*	0.06	0.12	13.84
*Microsporum canis*	0.21	>0.25	>3.31
*Microsporum gypseum*	0.08	>0.25	>6.65

**Table 3:** Cytotoxic concentrations required to achieve 50% cell death (CC_50/72h_) of oregano essential oil (OEO, % *v*/*v*), rosemary essential oil (REO, % *v*/*v*), and biogenic silver nanoparticles (bioAgNPs, µg/mL) after 72 h of treatment. The assay was conducted using Vero cells.

Active Compound	CC_50/72h_
OEO	>7.00
REO	0.05
bioAgNPs	2.26

Based on the density of the essential oils (0.9468 g/mL for OEO and 0.9130 g/mL for REO), the CC_50_ values for OEO and REO are >66 mg/mL and 1.46 mg/mL, respectively.

**Table 4:** Ex vivo antifungal efficacy of the formulations containing oregano (OEO), rosemary essential oil (REO), and biogenic silver nanoparticles (bioAgNPs) against various fungal species grown on nails. Three treatment durations (5 days, 10 days, and 15 days) were tested for each formulation. The base formulation (BF), which lacks active ingredients, was used as a control for fungal growth. For comparison, a commercial reference formulation (RF) was also evaluated. A positive sign (+) and green color indicate that the formulation exhibited antifungal efficacy, while a negative sign (−) and pale pink indicate that the formulation did not demonstrate antifungal efficacy.

Formulations	Duration of Treatment (Days)	Fungal Species			
		*Trichophyton mentagrophytes*	*Trichophyton rubrum*	*Microsporum* *canis*	*Microsporum gypseum*
F-OEO	5	+	+	*+*	+
	10	+	+	+	+
	15	+	+	+	+
F-REO	5	+	+	−	−
	10	+	+	+	+
	15	+	+	+	+
F-bioAgNPs	5	+	−	−	+
	10	+	+	−	+
	15	+	+	+	+
F-OEO/REO	5	+	+	+	+
	10	+	+	+	+
	15	+	+	+	+
F-REO/bioAgNPs	5	+	−	−	+
	10	+	+	+	+
	15	+	+	+	+
F-OEO/bioAgNPs	5	+	+	+	+
	10	+	+	+	+
	15	+	+	+	+
F-OEO/REO/bioAgNPs	5	+	−	−	−
	10	+	+	−	−
	15	+	+	−	+
RF	5	+	+	+	+
	10	+	+	+	+
	15	+	+	+	+
BF	5	−	−	−	−
	10	−	−	−	−
	15	−	−	−	−

**Table 5:** Pharmacotechnical characteristics of formulations containing OEO or OEO in combination with bioAgNPs.

Pharmacotechnical Characteristics	F-OEO	F-OEO/bioAgNPs	BF
Centrifuge test	NPSP	NPSP	NPSP
Aspect	homogeneous liquid	homogeneous liquid	homogeneous liquid
Color	slightly yellowish	translucent light brown	colorless
Odor	OEO	OEO	ethyl acetate
pH	4.0	4.4	4.4
Density (g/cm^3^)	9.99 ± 0.07	10.1 ± 0.01	9.91 ± 0.004
Drying time (s)	59.45 ± 1.98	90.30 ± 7.81	74.06 ± 2.21

NPSP: No Phase Separation or Precipitation; OEO: Oregano Essential Oil.

**Table 6:** Concentrations of active ingredients (%, *v*/*v*) in the formulated nail lacquers. Seven formulations containing oregano essential oil (OEO), rosemary essential oil (REO), and biogenic silver nanoparticles (bioAgNPs) were developed.

Formulations	OEO	REO	bioAgNPs
F-OEO	7	-	-
F-REO	-	7	-
F-bioAgNPs	-	-	2.5
F-OEO/REO	3.5	3.5	-
F-REO/bioAgNPs	-	3.5	1.25
F-OEO/bioAgNPs	3.5	-	1.25
F-OEO/REO/bioAgNPs	2.33	2.33	0.83
BF	-	-	-

The base formulation (BF) consists of cellulose acetate butyrate, isopropyl alcohol, ethyl acetate, and sucrose acetate butyrate. It is the same composition as the other formulations, which also contain active ingredients as specified in the table. It should be noted that 2.5%, 1.25%, and 0.83% (*v*/*v*) of bioAgNPs are equivalent to 4.25 µg/mL, 2.12 µg/mL, and 1.41 µg/mL, respectively.

## Data Availability

The original contributions presented in the study are included in the article/Supplementary Material, further inquiries can be directed to the corresponding authors.

## Supplementary Materials

The following supporting information can be downloaded at: https://www.mdpi.com/article/10.3390/antibiotics13090892/s1, Figure S1: Color analysis of the final formulations of the antifungal nail lacker; Table S1: Concentration unit conversion for oregano essential oil (OEO), rosemary essential oil (REO), and biogenic silver nanoparticles (bioAgNPs).
