# Supplementary material for: Nail Lacquer Containing Origanum vulgare and Rosmarinus officinalis Essential Oils and Biogenic Silver Nanoparticles for Onychomycosis: Development, Characterization, and Evaluation of Antifungal Efficacy
Source: Antibiotics (Basel). 2024 Sep 17;13(9):892. doi: 10.3390/antibiotics13090892 (PMC11428305; doi:10.3390/antibiotics13090892)
Supplement: Supplementary file 1 [file antibiotics-13-00892-s001.zip › antibiotics-3183357-supplementary.pdf]

antibiotics

Supplementary Material

This supplementary material contains one figure and one table.

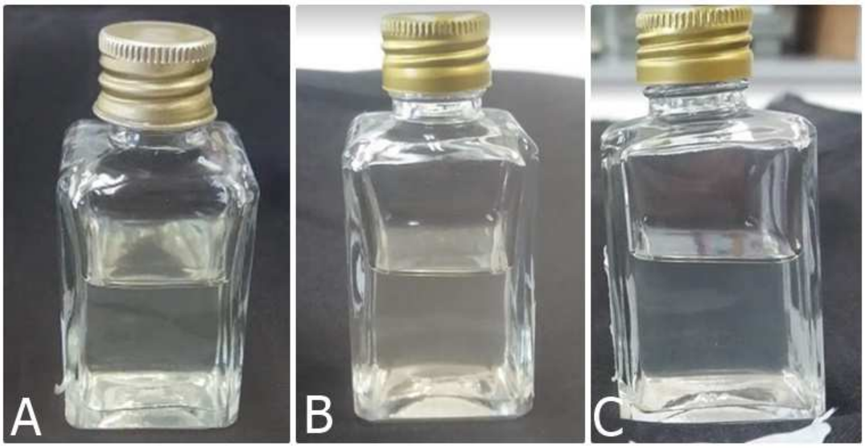

Figure S1. Final formulations of the antifungal nail lacker. **(A)** Base formulation without active ingredients (BF). **(B)** Formulation with oregano essential oil (F-OEO). **(C)** Formulation with oregano essential oil associated with biogenic silver nanoparticles (F-OEO/bioAgNPs).

Table S1. Concentration unit conversion for oregano essential oil (OEO), rosemary essential oil (REO) and biogenic silver nanoparticles (bioAgNPs).

OEO		REO		bioAgPs	
% (v/v)	mg/mL	% (v/v)	mg/mL	µM	µg/mL
10	94.68	10	91.30	250	42.50
5	47.34	5	45.65	125	21.25
2.5	23.67	2.5	22.82	62.5	10.62
1.25	11.83	1.25	11.41	31.25	5,31
0.62	5.92	0.62	5.71	15.62	2.66
0.31	2.96	0.31	2.85	7.81	1.33
0.16	1.48	0.16	1.43	3.91	0.66
0.08	0.74	0.08	0.71	1.95	0.33
0.04	0.37	0.04	0.36	0.98	0.16
0.02	0.18	0.02	0.18	0.49	0.08

Density was 0.9468 g/mL for OEO and 0.9130 g/mL for REO. The bioAgNP stock was at 1 mM.
